# Case Report: A case of haploinsufficiency of A20 in a newborn with abnormal liver enzymes at disease onset

**DOI:** 10.3389/fped.2026.1697285

**Published:** 2026-03-18

**Authors:** Yuanying Yu, Saizhen Zeng

**Affiliations:** Hunan Provincial People's Hospital and The First Affiliated Hospital of Hunan Normal University, Changsha, Hunan, China

**Keywords:** autoimmunity, HA20 syndrome, liver damage, newborn lupus, TNFAIP3

## Abstract

A20 haploinsufficiency (HA20) is an early-onset monogenic autoinflammatory disease caused by loss-of-function variants in the *TNFAIP3* gene, which encodes the A20 protein. Clinically, HA20 typically manifests with Behçet's disease-like features and often occurs in patients with a family history. Herein, we report a 1-month-old Chinese male infant who developed oral ulcers during the neonatal period, followed by recurrent fever, rash, elevated inflammatory markers, elevated liver enzymes, and positive lupus-related antibodies, with no relevant family history. Subsequent whole-exome sequencing identified a heterozygous variant in *TNFAIP3*: *c.1876_1877del (p. Leu626ValfsTer45)*, leading to the diagnosis of HA20. The patient's symptoms resolved completely with glucocorticoid monotherapy. We recommend that HA20 should be considered in the differential diagnosis of autoinflammatory disorders in infants and young children presenting with recurrent fever and rash, even in the absence of a family history of HA20.

## Introduction

**Figure 1:**
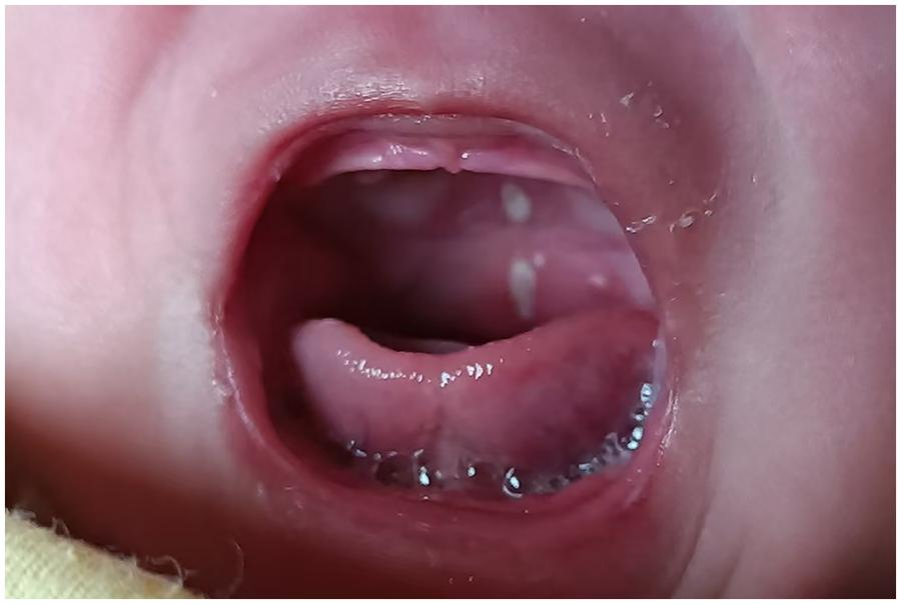
Oral ulcer in the child with A20 haploinsufficiency on day 22 after birth.

**Figure 2:**
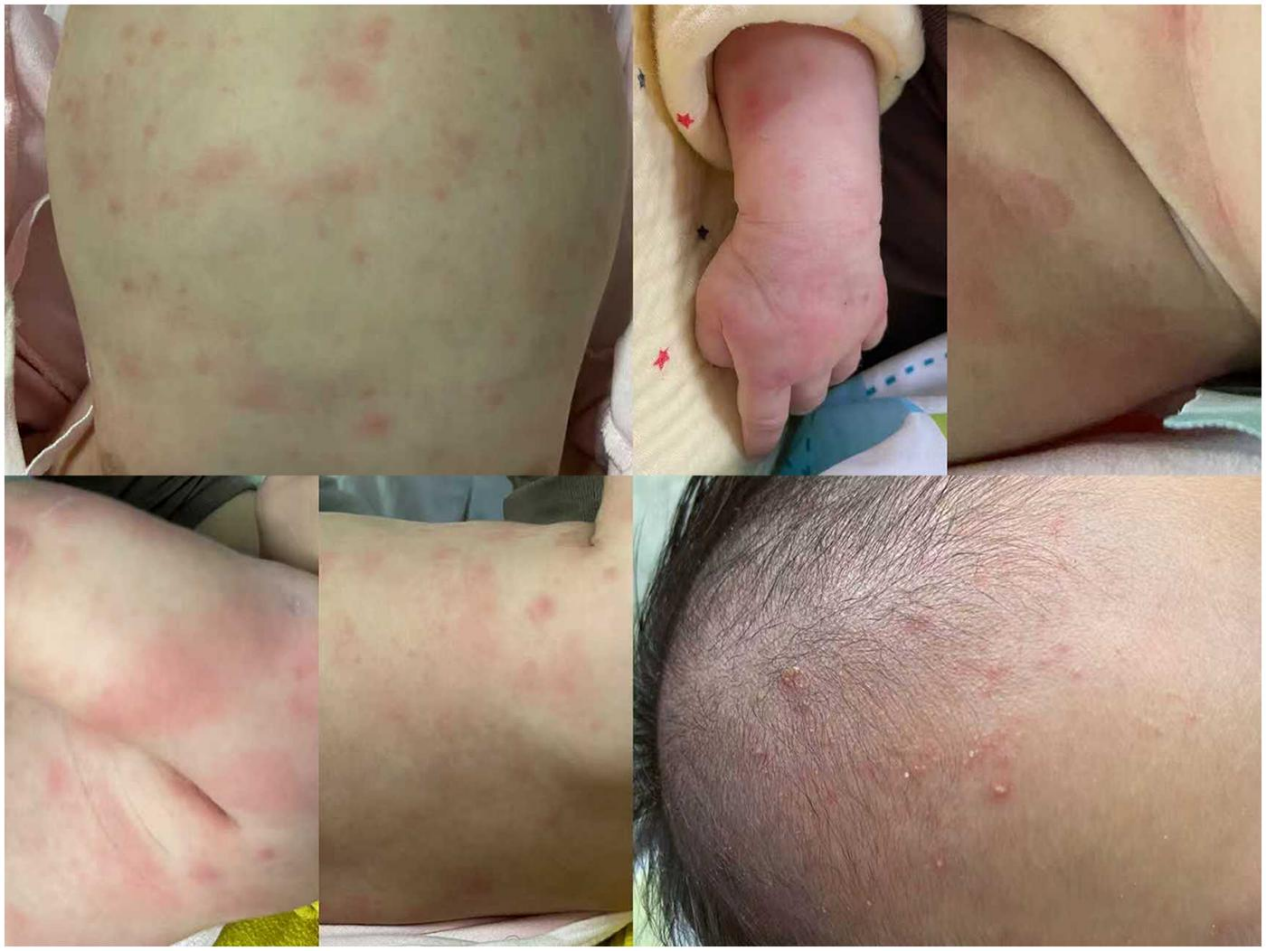
Rashes in the child with A20 haploinsufficiency on day 48 after birth.

A 48-day-old Chinese male infant was admitted with a 26-day history of ulceration, 10-day history of rash, and 1-day history of fever. The child was found oral ulcers for feeding refusal on day 22 after birth (Figure 1). A generalised erythematous rash appeared on postnatal day 38 (Figure 2) and persisted. Fever developed on postnatal day 47, with body temperature fluctuating between 38.5 ℃ and 39.5 ℃ (101.3–103.1℉), and the rash worsened during febrile episodes. The mother had an uneventful pregnancy and delivery. The infant was the father's first child, and his two half-brothers had no similar medical history. On admission, scattered erythematous rashes was observed across the entire body, these were blanchable and partially raised. The pharyngeal mucosa was congested, with two oral mucosal ulcers (approximately 5 mm in diameter) on the hard palate. Cardiopulmonary examination was normal. The child's abdomen was soft, with the liver palpable 2.5 cm below the costal margin and the spleen was palpable 1.5 cm below the costal margin. No abnormalities were detected in the anus or external genitalia. Laboratory findings are summarised in Table 1.

**Table 1:** Laboratory results of the child with A20 haploinsufficiency.

Project name	Value	Project name	Value	Project name	Value
<Peripheral blood>		<Biochemistry>		<Viral markers>	
White blood cell	**22.84** **×** **10^9^/L (4.3–14.2** **×** **10^9^/L)**	Total protein	60.8 g/L (49–71 g/L)	Influenza-A	(-)
Neutrophil	**64.9% (7–56%)**	Albumin	**33.2 g/L (35–50 g/L)**	Influenza-B	(-)
Monocyte	**16.6% (3–16%)**	Alanine aminotransferase	**153.1 U/L (8–71 U/L)**	Mycoplasma pneumoniae	(-)
Eosinophil	**0.9% (1–10%)**	Aspartate aminotransferase	**144.8 U/L (21–80 U/L)**	Adenovirus	(-)
Basophil	0% (0–1%)	Total bilirubin	4.42 umol/L (0–26 umol/L)	Human rhinovirus	(-)
Red blood cell	**4.10 × 10^12^/L (4.3–14.2 × 10^12^/L)**	Direct bilirubin	2.44 umol/L (0–6.1 umol/L)	RSV	(-)
Hemoglobin	**93 g/L (97–183 g/L)**	Total bile acid	14.6 umol/L (0–25 umol/L)	HIV Ag/Ab	(-)
Platelet	370 **×** 10^9^/L (183–614 **×** 10^9^/L)	Blood urea nitrogen	3.03 mmol/L (0.8–5.3 mmol/L)	Anti-TP	(-)
C-reactive protein	**61.33 mg/L (1–10 mg/L)**	Creatinine	3.03 mmol/L (0.8–5.3 mmol/L)	HBsAg	(-)
Procalcitonin	**0.27 ng/mL (0–0.06 ng/mL)**	Sodium	135.5 mmol/L (135–150 mmol/L)	Anti-HCV	(-)
Erythrocyte sedimentation rate	15 mm/h (1–15 mm/h)	Potassium	5.27 mmol/L (4.2–5.9 mmol/L)	CMV-DNA	(-)
<Immunology>		< Lupus antibodies >		Sputum culture	**Enterobacter cloacae**
immunoglobulin G	13.4 g/L (7–16 g/L)	MPO	**122.5R U/mL (0–20 R U/mL)**	Mycobacterium tuberculosis	(-)
immunoglobulin A	0.889 g/L (0.7–4 g/L)	PR3	**80.78R U/mL (0–20 R U/mL)**	< Direct antiglobulin test >	
Immunoglobulin M	1.03 g/L (0.4–2.3 g/L)	GBM	**186.01R U/mL (0–20 R U/mL)**	Coombs- IgG	(-)
Complement C3	**0.58 g/L (0.9–1.8 g/L)**	dsDNA-ELISE	**379.82I U/mL (1–100 IU/mL)**	Coombs- C3	**(++)**
Complement C4	**0.039 g/L (0.1–0.4 g/L)**	dsDNA-IIF	(-)	Coombs-Dott	**(++)**
<Cytokines>		pANCA	(-)	< Antiphospholipid antibody test >	
Interleukin-6	**181.58 pg/mL (0–6.62 pg/mL)**	cANCA	(-)	Cardiolipin-IgM	1.48MPL U**/**ML (<12MPL U**/**ML)
Interleukin-10	**2.77 pg/mL (0–2.31 pg/mL)**	Anti-Nuclear Antibody	**(+)**	Cardiolipin-IgG	10.4GPL U**/**ML (<12GPL U**/**ML)
TNF-ɑ	**41.31 pg/mL (0–33.27 pg/mL)**	Anti-Smith Antibody	(-)	Cardiolipin-IgA	0.39APL U**/**ML (<12APL U**/**ML)
Interferon-gamma	**31.51 pg/mL (0–20.6 pg/mL)**	SS-A/SS-B	(-)	Anti-glycoprotein antibody	**247.3R U/mL (0–20R U/mL)**

TNF-ɑ, tumor necrosis factor-alpha; MPO, anti-myeloperoxidase antibody IgG; PR3, anti-proteinase 3 antibody IgG; GBM, anti-glomerular basement membrane antibody IgG; dsDNA, anti-double-stranded DNA antibody(IgG type); ANCA, anti-neutrophil cytoplasmic antibody; Ab, antibody; Ag, antigen; RSV, respiratory syncytial virus; HIV, human immunodeficiency virus; TP, treponema pallidum; HBs, hepatitis B surface; HCV, hepatitis C virus; CMV, cytomegalovirus.

The bold values provided in Table 1 indicates abnormal results.

**Figure 3:**
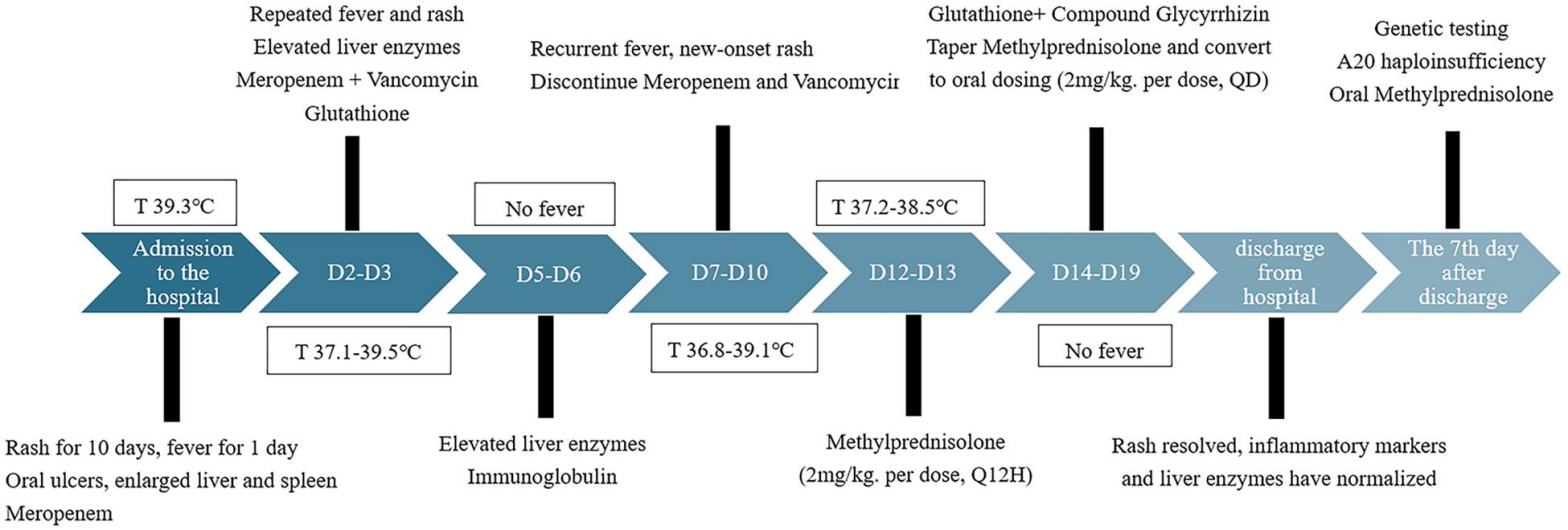
Flow chart of the first hospitalisation.

Laboratory tests revealed significantly elevated C-reactive protein (CRP) (61.33 mg/L) and white blood cell count (22.84 × 10⁹/L). After 3 days of anti-infective treatment with meropenem and vancomycin, patient's fever (37.1–39.5 ℃) and rash continued. Intravenous immunoglobulin (2 g/kg) was administered on day 4 of admission. After 2 days of normal temperature, fever recurred. Lupus-related antibody testing was positive for antinuclear antibodies, double-stranded DNA enzyme-linked immunosorbent assay, myeloperoxidase, proteinase 3, and glomerular basement membrane. Comprehensive metagenomic next-generation sequencing did not identify any pathogens. An autoinflammatory disease was therefore suspected, and whole-exome sequencing was performed. On day 14 of admission, intravenous methylprednisolone (4 mg/kg/day) was initiated. After 1 day, the patient's temperature normalised and the rash markedly improved. After 4 days of treatment, the oral ulcers had reduced in size, and the methylprednisolone dose was adjusted to 2 mg/kg/day. The patient was discharged on day 23 of hospitalisation (Figure 3).

Notably, liver enzymes were elevated on admission [alanine aminotransferase (ALT), 153.1 U/L; aspartate aminotransferase (AST), 144.8 U/L]. There was no hyperbilirubinaemia or bleeding tendency. Liver enzyme levels returned to normal after 4 days of glutathione therapy. However, when fever recurred on day 7 of admission, liver enzymes increased again (ALT, 234.8 U/L; AST, 136 U/L). There was no significant improvement following glutathione and glycyrrhizin hepatoprotective therapy. After initiation of methylprednisolone, liver enzyme levels decreased by day 5 and returned to normal by day 9 of treatment.

The child also developed intermittent diarrhoea lasting approximately 7 days during hospitalisation, with three to five episodes of yellow-green loose stools per day and no mucus or blood. Stool culture for Salmonella and enterovirus nucleic acid testing were negative. The diarrhoea resolved after 4 days of methylprednisolone treatment.

**Figure 4:**
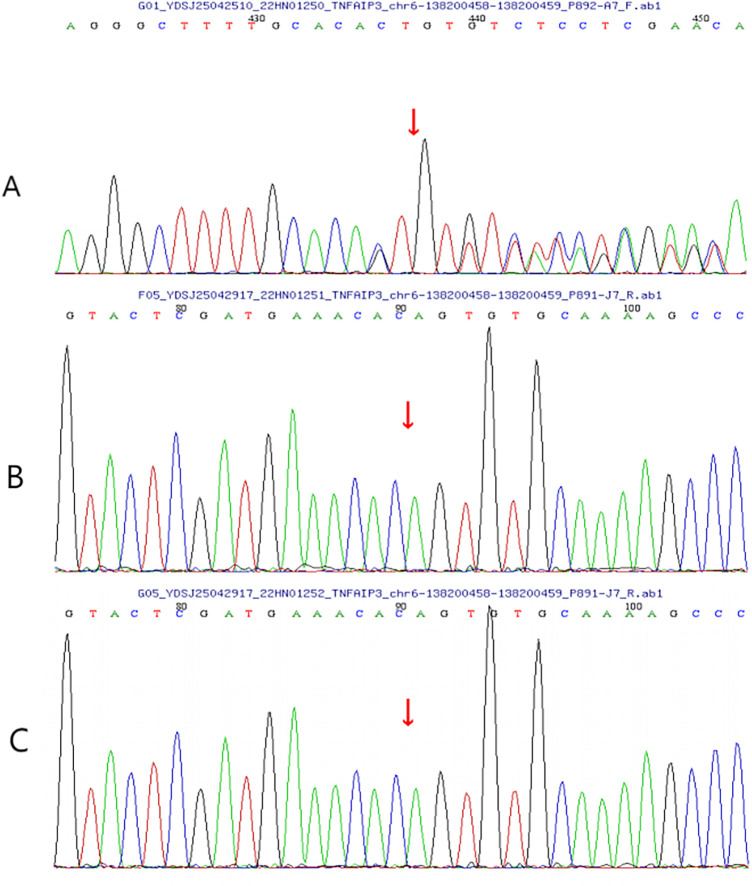
TNFAIP3 chr6:(138200458-138200459), NM_001270508.2c.1876_1877del (p.Leu626ValfsTer45). **(A)** The child's DNA sequencing chromatograms. **(B)** The DNA sequencing chromatograms of the child's father. **(C)** The DNA sequencing chromatograms of the child's mother.

On day 3 after discharge, the child was readmitted with fever and diarrhoea. CRP (166 mg/L) and procalcitonin (19.22 ng/mL) were elevated, and COVID-19 nucleic acid testing was positive. After 3 days of supportive therapy and intravenous methylprednisolone (2 mg/kg/day), the child's temperature normalised and CRP levels returned to normal. The methylprednisolone dose was reduced to 1.5 mg/kg/day on day 4 of readmission. Diarrhoea resolved after 7 days. During this hospitalisation, whole-exome sequencing results became available, revealing a heterozygous mutation in the *TNFAIP3* gene at *chr6:138200458–138200459, NM_001270508.2:c.1876_1877del (p.Leu626ValfsTer45)*. Sanger sequencing confirmed that neither parent carried the variant (Figure 4), indicating a *de novo* frameshift mutation. According to the American College of Medical Genetics and Genomics guidelines, the variant was classified as pathogenic (PVS1 + PS_Moderate + PS4_Supporting + PM2_Supporting). Based on this heterozygous *TNFAIP3* mutation, the patient was diagnosed with A20 haploinsufficiency (HA20). Because no new rashes or oral ulcers developed and inflammatory markers normalised, biologic agents were not administered. The patient continued oral methylprednisolone after discharge, with gradual dose reduction.

The patient was followed for 8 months. During this period, he experienced eight hospitalisations with fever and elevated inflammatory markers. No new rashes or oral ulcers occurred, and liver enzymes remained normal. Diarrhoea occurred during seven hospitalisations, with enterovirus detected in only one episode. At the time of this writing, the patient had been asymptomatic for 2 months while receiving maintenance methylprednisolone (0.5 mg/kg/day) and he remained under regular follow-up. However, growth restriction was noted at 4 months of age, with height (56 cm) and weight (5.5 kg) below the third percentile for age and sex. At 10 months of age, height was 65 cm and weight was 6.5 kg.

## Discussion

HA20 was first described by Zhou (1) in 2016. It is a monogenic autoinflammatory disease caused by variants in the *TNFAIP3* gene, leading to dysregulation of the nuclear factor kappa-B (NF-κB) signalling pathway. A20 consists of an N-terminal ovarian tumour domain followed by seven zinc finger domains, conferring both ubiquitination and deubiquitination functions. Through its N-terminal ovarian tumour domain, A20 removes K63-linked ubiquitin chains and adds K48-linked ubiquitin chains, thereby regulating protein stability and degradation. This dual activity plays a critical role in inhibiting NF-κB activity, suppressing mitogen-activated protein kinase (MAPK) activation, and downregulating NOD-like receptor family pyrin domain containing 3 (NLRP3) inflammasome activation (2, 3). Consequently, the inflammatory manifestations of HA20 are attributed to insufficient inhibition of NF-κB, MAPK, and NLRP3 pathways, resulting in excessive production of pro-inflammatory cytokines such as interleukin (IL)-1β, IL-6, IL-18, and tumour necrosis factor-alpha (TNF-α). In this patient, inflammatory cytokines (including IL-6, IL-10, TNF-α, and interferon-*γ*) were elevated, consistent with inadequate suppression of inflammatory signalling.

The onset of HA20 ranges from the neonatal period to approximately 30 years of age (4). Patients with larger gene deletions often present in early infancy, with more than 80% developing symptoms within the first year of life (5). Reported infant cases commonly present with fever, mucosal ulcers, positive autoantibodies, and gastrointestinal involvement (6, 7). HA20 was a multisystem inflammatory disease, with clinical manifestations including fever (approximately 50% of cases), recurrent urogenital ulcers (>70%), gastrointestinal involvement (serositis, gastrointestinal ulcers, small bowel inflammation, or inflammatory colitis, as well as a risk of intestinal perforation in severe cases), cutaneous lesions (pustular rash, folliculitis, vasculitic purpura, urticaria, lupus-like macules, and eczematoid dermatitis), and autoimmune diseases such as systemic lupus erythematosus and Behçet's disease (8). It may also be accompanied by immunodeficiency (hypogammaglobulinaemia, common variant immunodeficiency) (9). Additional features include respiratory infections, chronic liver disease, vascular lesions, ocular inflammation, and neurological involvement (10). In this case, disease onset occurred in the neonatal period, with fever, rash, oral ulcers, diarrhoea, and positive autoantibodies, consistent with previously reported clinical features. Given the recurrent diarrhoea, gastroscopy and colonoscopy were recommended, but the patient's father declined. The patient remains under ongoing follow-up.

Elevated liver enzymes in HA20 have received limited attention. Previous studies have reported liver involvement in approximately 8% of patients, although the underlying mechanism remains unclear (11). Iwasa (12) described a 33-year-old woman with HA20 who was diagnosed in childhood. She presented with elevated liver enzymes and jaundice and was diagnosed with autoimmune hepatitis by liver biopsy. Zhang (7) reported a case of HA20 with liver injury that had onset in the neonatal period; however, in that case, cytomegalovirus nucleic acid testing was positive. In the present case, elevations of liver enzymes were observed during the first hospitalisation, and no pathogen was detected. This suggests that elevated liver enzymes may represent one of the clinical manifestations of HA20. Liver function was normal during follow-up, and liver biopsy was not performed. A previous study (13) confirmed that the A20 protein is a major hepatoprotective factor under inflammatory and cytotoxic conditions in mice. Specific mutations or polymorphisms in the A20 locus may increase susceptibility to chronic liver diseases, such as alcoholic liver disease, non-alcoholic fatty liver disease, and viral hepatitis—conditions known to promote the development of cirrhosis and hepatocellular carcinoma. These findings suggest that patients with HA20 should undergo regular monitoring of liver function.

At present, HA20 lacks specific treatment guidelines and remains incurable, with supportive care as the primary management approach. Reports have confirmed that glucocorticoid therapy is effective in HA20, as in other autoimmune inflammatory diseases, although some cases require biologic agents to control symptoms (14). TNF inhibitors directly target aberrant NF-κB signalling and are commonly used in the management of HA20. Other therapeutic options include colchicine, methotrexate, azathioprine, thalidomide, and mefenamic acid. Haematopoietic stem cell transplantation is also an option for severe or refractory cases (7). In the present case, the patient's symptoms were well controlled with glucocorticoid monotherapy. Delayed growth and development were noted during follow-up, highlighting the need for ongoing monitoring of disease progression and close communication with the parents.

## Summary

HA20 may manifest with rash, oral ulceration, diarrhoea and can be accompanied by positive autoantibodies. It requires differentiation from systemic lupus erythematosus and Behçet's disease. In infants with neonatal-onset disease accompanied by fever, rash, diarrhoea, and elevated inflammatory markers, HA20 should be considered even in the absence of a family history. Timely completion of whole-exome sequencing is crucial for the definitive diagnosis of HA20. For patients with a confirmed diagnosis of HA20, regular monitoring of liver function is required, and close attention should also be paid to growth and development.

## Data Availability

The original contributions presented in the study are included in the article/supplementary material, further inquiries can be directed to the corresponding author/s.
